# Microfluidic and Turbulent Mixing for mRNA LNP Vaccines

**DOI:** 10.3390/pharmaceutics17091148

**Published:** 2025-09-01

**Authors:** Patrick L. Ahl

**Affiliations:** PLA Formulation Consulting LLC, Princeton, NJ 08540, USA; brpat74@gmail.com

**Keywords:** nanoparticle, vaccines, mRNA, LNP, microfluidic, turbulent

## Abstract

Using lipid nanocarriers to deliver the mRNA of a specific antigen to immune cells is a powerful innovative approach to rapidly develop new safe and effective vaccines. Understanding and optimizing the mixing process necessary for mRNA lipid nanoparticles (LNPs) is the focus of this review. The first objective is to review the fundamentals of microfluidic and turbulent fluid-mixing basics needed to understand the mixing process. The mRNA LNP self-assembly flash nanoprecipitation/self-assembly process will be discussed. Then, some important experimental nanoparticle studies which are the basis for the current understanding of microfluidic and turbulent mRNA LNP mixing process will be reviewed. Finally, the current commercially available LNP mixing technology will be summarized. There appears to be no universally “best” mixing process for formulating nanoparticles or mRNA LNPs. Both chaotic advection and turbulent flow microfluidic mixing devices, using the proper parameters for each device, will formulate similar mRNA LNP vaccines during development research. However, the low fluid output of microfluidic devices may not be practicable at higher fluid flow rates. Larger-scale turbulent mixing devices are more suitable for clinical-scale mRNA LNP production.

## 1. Introduction

Small-molecule agonists or antagonists to specific sites on specific enzymes or receptors were the primary focus of pharmaceutical activity in the 20th century. The delivery of these bioactive molecules to cellular sites is typically accomplished by passive diffusion and permeation through membranes. The medical opportunities in the 21st century demand moving beyond inefficient non-targeted passive delivery of small bioactive molecules. These new medical opportunities require controlled in vivo release of small bioactive molecules and intracellular delivery of unstable biomolecules, such as plasmid DNA (pDNA), small interfering RNA (siRNA), and messenger RNA (mRNA). These 21st century demands require the development of nanocarriers for delivery of both bioactive molecules and vaccines. This has resulted in an explosion of biocompatible nanoparticle research, development, and clinical application. Sustained in vivo release of small bioactive molecules is the application of many long-acting injectable (LAI) nanoparticle formulations. Formulation of biocompatible nanoparticles for therapeutic LAI is outside the scope of this review [1,2].

The primary focus will be on fluid-mixing technology used to formulate lipid nanoparticles (LNPs) to encapsulate nucleic acids such as pDNA, siRNA, and mRNA. The emphasis of this review will be on fluid-mixing issues related to mRNA LNP formation because of the high importance of mRNA LNP vaccine development at this time. A full evaluation of all aspects of mRNA LNP vaccine efficacy and safety is beyond the scope of this review. Amphiphilic lipids such as phospholipids that can form lyotropic liquid-crystalline mesophases are required for LNP self-assembly. The LNP amphiphilic lipids must be at the appropriate concentration and temperature for assembly into the proper liquid-crystalline phase. A liquid-crystalline phase can behave like a liquid or solid crystal [3].

“Self-assembly” is a process in which a disordered system of pre-existing components form an organized structure or pattern because of specific local interactions among the components themselves, without external direction [4]. The level of structural organization is enough in the nanoparticle to maintain a thermodynamically stable diameter. Even so, individual biomolecules within the structure may not be highly organized relative to each other, i.e., a liquid-crystalline phase. Our working definition of nanoparticles will be an aqueous suspension of self-assembled lipid/nucleic acid nanoparticles with a weight-averaged diameter in the 10 to 500 nm range. Natural self-assembly is essentially the process responsible for producing macromolecular structures within all living cells, such as cell membranes and ribosomes [5]. Even protein folding itself can be considered a form of self-assembly [6]. Pharmaceutical scientists have used self-assembly to prepare therapeutically efficacious nanoparticles for drug delivery [7], medical diagnostic and imaging [8], vaccine antigen [9], and vaccine adjuvant [10] delivery.

Lipid nanoparticle (LNP) formulation methods involve nanoprecipitation followed by self-assembly of lipids and nucleic acids. This review will focus on the solvent/antisolvent mixing process, followed by the self-assembly process that has been used to prepare liposomes, oil–water emulsions, and many other various LNP formulations [11]. The two FDA-approved COVID-19 mRNA LNP vaccines are formulated by solvent/antisolvent mixing and self-assembly [12,13]. This liposome and LNP self-assembly process typically requires a critical rapid mixing step where a miscible organic solvent stream (solvent) with dissolved hydrophobic lipid molecules and aqueous stream (anti-solvent) with water-soluble molecules are combined. Mixing nanoprecipitation occurs when the hydrophobic lipid molecules get rapidly solvent-exchanged into a more hydrophilic mixed solvent. This causes lipid molecules to coalesce into lyotropic liquid-crystalline self-assembled lipid structures like micelles, bilayers, and hexagonal phases. In addition, ionizable cationic lipids are added to the miscible organic solvent to form electrostatically associated nanoparticles with poly-anionic biomolecules like pDNA, siRNA, and mRNA to promote nucleic acid-encapsulated LNPs. The mRNA LNP solvent-exchange formulation process and mixing technology has moved to the center of 21st-century vaccine development [14,15]. The rapid development of mRNA LNP vaccines for the COVID-19 pandemic is the premier example of the importance nanoparticle technology to vaccine development and public health [16,17]. This review will focus primarily on the mixing process needed to obtain homogenous self-assembled for mRNA LNP that can be used for mRNA LNP vaccines. It will present basic equations of microfluidic and turbulent flow mixing, along with some recent mRNA LNP formulation research. The commercially available microfluidic and turbulent mixing devices used to formulate mRNA LNPs by the solvent and non-solvent stream mixing will be presented. Many of the critical specific biochemical details of mRNA modification and ionizable cationic lipid options for mRNA LNP vaccine development are discussed in other reviews [18,19].

## 2. Fundamentals of Diffusion-Mediated Mixing Distances

The purpose of fluid mixing is to achieve uniform concentrations of all chemical components throughout the mixing container. In most situations, the final structure and size of a nanoparticle will have a strong dependence on the local self-assembly environment during the assembly time of the nanoparticle. Homogeneous chemical concentrations throughout the mixing container during self-assembly are often very important for uniform nanoparticle assembly throughout the mixing container. When the local self-assembly chemical environment is not homogeneous, the nanoparticle self-assembly process will not be homogeneous throughout the mixing container. Ultimately, molecular diffusion is the process which completes the homogenization of all the molecules in the fluid-mixing chamber. Nanoparticle self-assembly time should be significantly longer than time for diffusion to homogenize the concentrations of the mixed fluids. Fick’s second law of diffusion predicts how diffusion causes concentrations to change with respect to time [20]. Fick’s second law can be used to calculate the Brownian motion Mean Squared Displacement (MSD) from the original position for diffusing particles (Equation (1)):

(1) $$MSD={\langle x-x_{0}\rangle }^{2}=2nDt$$

where

$\langle x-x_{0}\rangle =$ Average displacement distance
n= dimension
D= diffusivity t= time

The average distance that molecules must diffuse to homogenize a concentration is called the striation length $l_{st}$. The homogenization mixing time ($t_{mix}$) for a given striation distance, $l_{st},$ is proportional to ${l_{st}}^{2}$ divided by D (Equation (2)):

(2) $$t_{mix}\;=\;\frac{{l_{st}}^{2}}{2D}$$

**Figure 1:**
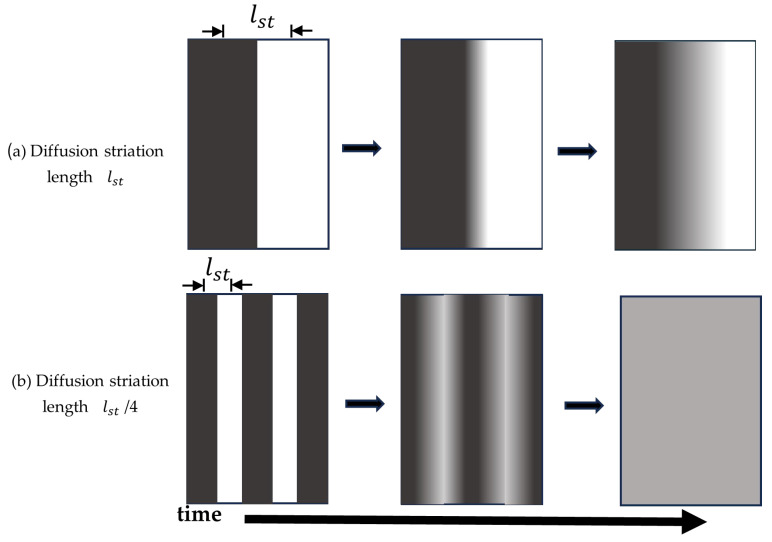
Illustration of how decreasing striation length ($l_{st}$) reduces diffusion mixing time ($t_{mix}$). $l_{st}$ is the distance that diffusion must occur to homogenize two solutions. (**a**) An unmixed solution with $l_{st}$ = 1 distance. (**b**) An unmixed solution with $l_{st}$ = ¼ distance. The $l_{st}$ = 1 solution is not mixed after time, while $t_{mix}$ for the $l_{st}$ = ¼ distance solution is reduced by 16-fold.

Molecular diffusion over macroscopic mixing distances is a slow process compared to estimated nanoparticle self-assembly times. For example, even for a small molecule like sucrose diffusing in water with D= 10−9 m2s, the average time to diffuse a $l_{st}$ of 1 mm in would be over 8 min [21]. In most cases, nanoparticle self-assembly is estimated to occur on the timescale of a few milliseconds (msec) [22]. According to the $t_{mix}$ equation, homogenous mixing at a msec timescale requires $l_{st}$ distances of under 5 μm. Thus, reducing $l_{st}$ in the mixing device will decreasing fluid $t_{mix}$, which will improve the mixing efficiency. Reducing $l_{st}$ can be accomplished during a mixing process by stretching/folding and or breakup/rejoining adjacent mixing-fluid streams. These processes will decrease $l_{st}$, which will increase the contact area between adjacent fluids that are being mixed [23]. These fluid-stream procedures allow for molecular diffusion to quickly homogenize the solution concentrations. A simple illustration of how reducing ($l_{st}$) improves process mixing time is shown in Figure 1.

Mixers that rely solely on only pressure gradients to drive the fluids through elements such as mixing channels and chambers to achieve fluid mixing are referred to as passive mixers. Passive mixers are continuous flow systems which are preferred for scale-up and manufacturing. Mixers that mix contained static volumes, i.e., batches, of fluid by rapid stirring are considered “active” mixers. The review will not consider active mixers or other types of mixers which employ external energy, such as ultrasound or electro/magnetic fields [24]. This review will consider two classes of passive mixers: (1) microfluidic mixers and (2) turbulent flow mixers.

## 3. Microfluidic Mixer Fundamentals

**Figure 2:**
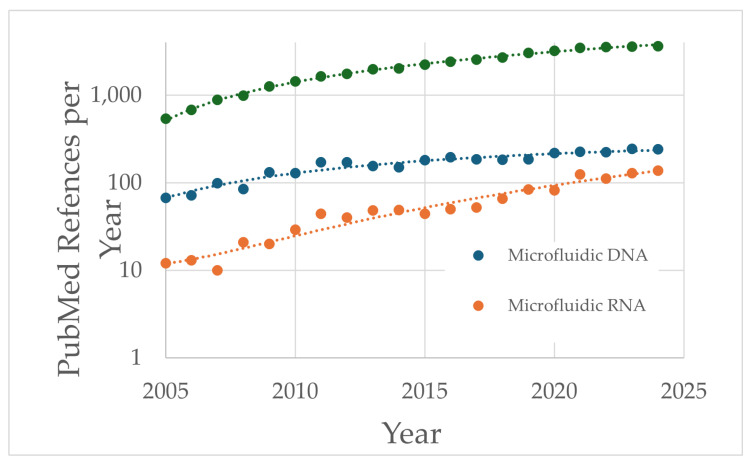
PubMed search results for the last 20 years for total microfluidics references (●), DNA microfluidics references (●), and RNA microfluidics references (●).

Microfluidic mixing was developed to achieve rapid and thorough mixing of multiple fluid steams of fluids in microscale devices [23]. The fluid channels and mixing-chamber diameters in microscale devices range up to a few hundred µm. Uniform diffusion mixing is easier to achieve operating at these diminutive mixing scales. Advanced microelectromechanical system (MEMS) technology has allowed for the fabrication of sophisticated microfluid chips for a wide range of applications, including “lab-on-a-chip” systems [25,26,27]. Numerous microfluidic mixer designs have been proposed and evaluated over the last two decades for many applications. The focus in this review will be on vaccine nucleic acid LNP applications. The popularity of microfluidic biotech technology and for DNA and RNA applications, as indicated by PubMed references, has steadily increased during the last 20 years (Figure 2).

Simple laminar-flow microfluidic mixing devices use the slow process of diffusion to mix thin adjacent fluid streams over relatively long microchannel distances. Most microfluidic mixers used for mRNA LNP formulations have been designed with features to increase the mixing of fluid contact areas by fluid advection. This reduces $l_{st}$ between the mixing fluids by multiple folding of different fluid streams along the mixing channel. The fluid advection mixing induced by folding and bending fluid streams is often referred to as chaotic advection. This mixing behavior is characterized by recirculation zones in the mixing pathway which increase advection fluid mixing. Chaotic advection is usually produced by inserting properly designed obstructions in the mixing channel, such as herringbones or toroidal rings. These features are used in both the NanoAssemblr™ Benchtop™ and NanoAssemblr™ Ignite™ (Cytiva, Marlborough, MA, USA) microfluidic systems to improve the mixing efficiency [28]. The mRNA LNP formulation by microfluidic or turbulent mixing procedures produces a rapid solvent-exchange process. Neutral and ionizable cationic lipids are dissolved in a miscible organic fluid or “solvent”, which is often ethanol (${fl}_{eth}$), while the water-soluble components of the nanoparticle, such as mRNA, are dissolved in the aqueous pH buffered “anti-solvent fluid” (${fl}_{aqu}$). Both the microfluidic and turbulent mixing processes are designed to simply mix the ${fl}_{eth}$ with ${fl}_{aqu}$ at flow ratios such that the ethanol lipids are not soluble in the homogenized ethanol/water mixture as single molecules. The turbulent mixing process will be described in Section 4. A lyotropic mesophase is formed as the lipids and mRNA self-assemble to form a mixture of nanoparticles within the ${fl}_{aqu}$/${fl}_{eth}$ solution mixture. After the nanoparticles are formed by microfluidic or turbulent mixing, the ethanol content in the nanoparticle suspension is typically reduced by dialysis or tangential flow filtration (TFF). Reducing the ethanol concentration will induce additional rearrangements in the post-mixing particles, resulting in the final mRNA LNP structures [29]. This mRNA RNA post-mixing self-assembly process will be discussed in greater detail in Section 5.

A low Reynolds number ($R_{e}$) is a key feature of most microfluidic mixers. The dimensionless $R_{e}$ for any moving fluid is the ratio of the fluid inertia force divided by the viscous force (Equation (3)):

(3) $$R_{e\;}=\;\frac{\rho ul}{\mu }\;=\;\frac{ul}{\upsilon }$$

where

ρ = fluid density ($Kg/m^{3});$
u = fluid flow (m/s);
l = characteristic length (which is typically the microfluidic channel height);
μ= dynamic viscosity (Kg/ms);
υ= kinematic viscosity ($m^{2}/s$).

This equation can be used to estimate the $R_{e}$ for most microfluidic mixing systems used to prepare mRNA LNP by mixing an ethanol stream with lipids with an aqueous stream with mRNA. The characteristic length or channel height, l, of the mixing channels in this system is typically about 150 µm. The fluid flow rates at output are typically set between 0.2 and 12 mL/min. So, the calculated $R_{e\;}$ for most microfluidics system is between 8 and 800 depending on the fluid flow rate [30]. There is no sharp transition from laminar to turbulent fluid flows. Turbulent flow will be discussed in Section 5. Generally, $R_{e}\;>5000$ is considered completely turbulent, while $R_{e}$ values between 2000 and 5000 are often a mixture of laminar and turbulent flow. So, turbulent fluid mixing would not exist in this microfluid mixer because $R_{e}$ is <2000. Turbulent fluid mixing, i.e., $R_{e}>5000$, is difficult to achieve in microfluidic system because of the small channel dimensions and low fluid flow rates typically used in most microfluid technology,

The relative importance of chaotic advection mixing to slower diffusion mixing is characterized by the Peclet number ($P_{e\;}$), which is the ratio of the characteristic diffusion time (tdif =lst2D) to the characteristic chaotic advection convection time (tcon= lstμ) for the specified $l_{st}$ of the mixing channel (Equation (4)):

(4) Pe = lst2Dlstu = tdiftcon= lstuD

$P_{e}$ can be easily calculated in a microfluid mixer if the fluid’s $l_{st}$ and u are known, along with the diffusion constant, D, of the molecules being mixed. ${\;P}_{e}$ is useful because it can be used to estimate the required length of a microfluid-mixing channel, $l_{mix}$ if the $l_{st}$ of the channel mixing process is known or can be estimated (Equation (5)):

(5) $$t_{mix}\;=\;t_{con}\;P_{e}=\;\frac{l_{st}}{u}\;P_{e}$$

In the special case where the channel flow is completely laminar, then all mixing in the channel is due to diffusion. In this situation, the “striation length”, $l_{st}$, for the channel is just the height of the channel, l, meaning that there is no convection mixing, and the channel characteristic mixing time, $t_{mix},$ is equal to the characteristic diffusion mixing time, $t_{dif}$ (Equation (6)):

(6) $$t_{mix}\;\;=\;\;t_{dif}\;=\;t_{con}\;P_{e}$$

Since $t_{con}=\;\frac{l}{u}$ for the diffusion-only case, the microfluid channel’s $P_{e}$ can be used to estimate the microfluidic channel length, $l_{mix}$, required to obtain complete mixing by diffusion mixing alone. This is given by a simple equation (Equation (7)):

(7) $$l_{mix}=ut_{mix}=lP_{e}$$

In most microfluid fluid-mixing situations without chaotic advection, $P_{e}=\;\frac{lu}{D}$, will be too large when diffusion mixing only dominates the process. An excessively large $P_{e}$ results in the mixing channel length, $l_{mix}$, being too long for most practical microfluidic mixers. For example, if microchannel height, l, is 100 µm and the fluid flow rate is u = 1 cm/s, then the channel length required for diffusion mixing, $l_{mix}$, would need be 10 cm for a small molecule at D=10−9 m2s with $P_{e}=1000$. However, for a large biomolecule at D= 10−11m2s with $P_{e}=100,000$, the mixing channel would need to be 100 times greater at 10 m. A 10-m-long mixing channel is way too long for any practical microfluidic mixer. The solution to this excess length of $l_{mix}$ is to significantly reduce the $P_{e}$ value by significantly reducing the striation length ($l_{st}$). This is achieved by placing obstructions in the channel flow to introduce chaotic advection.

**Figure 3:**
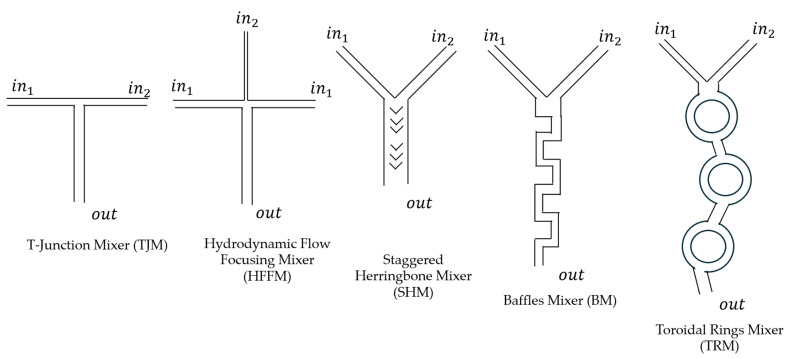
Five simple drawings of common microfluidic mixer designs. The input ports for mixing fluids are designated as ${in}_{1}$ and *in*_2_, while the combined fluid output port is designated as *out*.

Chaotic advection can be introduced by placing designed obstructions in the mixing channel, such as herringbones, baffles, or toroidal rings. Incorporating these features into microfluidic mixers will reduce $l_{st}$ and $P_{e}$, thus shorting the $l_{mix}$ required for complete mixing. The schematic channel designs of five common microfluidic mixers designed to increase chaotic advection flow and to decrease $l_{st}$ and $P_{e}$ are shown in Figure 3. Each of these designs has been used to prepare lab-scale mRNA LNP formulations. The general features of the five commonly used lab-scale microfluid mixers designed are shown in Figure 3 and discussed below.

### 3.1. T-Junction Mixers (TJMs)

These designs are the simplest mixing methods to rapidly mix two streams to prepare any type of LNP. In many microfluidic designs, low $R_{e}$ fluid streams, ${fl}_{eth}$, and ${fl}_{aqu}$ collide at a T-junction. This mixing arrangement is very simple to fabricate with small-diameter, “off-the-shelf” lab tubing. So, this mixer design is often used in early-stage LNP development program R&D and scale-up. The streams must collide with significant momentum to achieve chaotic advection mixing. However, under some conditions, the colliding fluid streams may partially deflect each other and then separately travel down the output channel. This will significantly increase the mixing time, unless suitable obstructions in the outlet channel are introduced to produce chaotic advection mixing. Lab-scale T-junction mixer LNP results can be non-reproducible and difficult to scale up. Increasing both input fluid velocities, u, to turbulent velocity is another way to improve mixing if the T-junction channels can accommodate the high fluid velocities, i.e., $R_{e}$. If the T-junction fluid $R_{e}$ ≥ 2000 the mixing might be turbulent, while $R_{e}$ ≤ 1000 the mixing would be chaotic advection. Ultimately, the mixing can contain elements of both chaotic advections mixing and turbulent mixing depending on the $R_{e}$ of colliding fluid stream. The complex mixing behavior with a strong dependance on u makes simple lab-scale T-junction mixing results irreproducible and often quite difficult to scale up.

### 3.2. Hydrodynamic Flow Focusing Mixers (HFFMs)

These designs are a popular method to prepare lab-scale and manufacture-scale quantities of drug nano-emulsions [31]. The ${fl}_{eth}$ stream is injected into the center of the channel at a low flow rate. Two symmetric ${fl}_{aqu}$ streams are injected at a higher flow rate along the sides of the ${fl}_{eth}$ central stream. The two symmetric ${fl}_{aqu}$ streams compress the central ${fl}_{eth}$ stream and reduce $l_{st}$ for the molecular diffusion required for mixing. HFFM devices can produce turbulent flow conditions by significantly increasing the central stream flow rate [32].

### 3.3. Staggered Herringbone Mixers (SHMs)

This microfluidic mixing design is used in the very popular NanoAssemblr™ Benchtop™ (Cytiva, Marlborough, MA, USA) developed by Precision Nanosystems LLC (Vancouver, BC, Canada) for mRNA LNP formulation [28]. This microfluidic mixing cartridge has a Y-junction input of the ${fl}_{eth}$ and ${fl}_{aqu}$ streams. Then, the fluid stream passes through a mixing channel with herringbone obstructions to promote chaotic advection that improves mixing efficiency. The company which now sells the NanoAssemblr™ (Cytiva, Marlborough, MA, USA) instrument technology has discontinued selling this SHM mixing cartridge [28].

### 3.4. Baffles Mixers (BMs)

These designs are common in many lab-designed microfluidic microchannels [33,34]. Sharp turns in the mixing channel promote backflow and recirculation, which cause the ${fl}_{eth}$ and ${fl}_{aqu}$ input streams to separate and fold over on each other, causing chaotic advection and, thus, reducing the mixing $l_{st}$. Large baffle dimensions and large fluid flow rates will promote larger recirculation zones and better mixing. However, baffles may also increase the probability of channel clogging at higher fluid flow rates depending on the components in the mixed fluids.

### 3.5. Toroidal Ring Mixers (TRMs)

These designs are a novel approach to increase chaotic advection in the mixing channel at a higher fluid flow rates than baffle designs without a large increase channel clogging. A toroidal ring mixing design is used in the Cytiva NxGen™ (Cytiva, Marlborough MA USA) microfluidic mixing device in the popular NanoAssemblr™ Ignite™ (Cytiva, Marlborough, MA, USA) mRNA instrument often used for lab-scale mRNA LNP vaccine formulation [28]. Toroidal ring microfluidic mixers have flow channels connected to channel rings, as shown in Figure 3. Splitting the ${fl}_{eth}$ and ${fl}_{aqu}$ input streams when the stream is entering a channel ring produces chaotic advection. In addition, there are fluid vortexes in the fluid produced by centrifugal forces of a fluid flowing through the curved channels of the toroidal rings. These vortexes also reduce $l_{st}$ in the fluids moving around the rings. The fluid flows through a curved channel can be characterized by a dimensionless value like $R_{e}$, called the Dean number, $D_{e}$ (Equation (8)).

(8) $$D_{e}=R_{e}\sqrt{\frac{d}{2r}}$$

where

$R_{e\;}$ = Reynold number of the fluid flow;
d= channel diameter;
r= radius of ring.

Like $R_{e}$, the $D_{e}$ value can characterize the fluid flow behavior in the curved channels in the rings. $D_{e}$ < 60 indicates laminar fluid flow in the ring. A $D_{e}$ value between 75 and 200 implies stable Dean vortexes in the curved channels, which produce chaotic advection. Meanwhile, $D_{e}\;$> 400 indicates complete turbulent flow in the curved microfluid mixing [30].

## 4. Turbulent Flow Mixer Fundamentals

The purpose of fluid mixing is to rapidly achieve uniform concentrations of all chemical components throughout the mixing container. The microfluidic mixing channels rely on chaotic advection to reduce $l_{st}$ to the level where molecular diffusion can complete the mixing process faster than the self-assembly time. Fluid turbulence is another fluid dynamics mechanism for reducing $l_{st}$ and, thus, $t_{mix}$ via the production of fluid eddies and vortexes. Characterization of turbulent flow has been considered one of the most important and complex problems in both physics and engineering for many years. An good simple introduction to the complex topic of fluid turbulence by trusted academics can be found on YouTube [35]. The fluid conditions of inertial fluid flow momentum and fluid viscosity that produce turbulent flow have been well recognized for over 100 years [36]. The balance between the fluid inertial force and the fluid viscous force is characterized by $R_{e},$ which helps determine whether the fluid flow is laminar or turbulent. $R_{e}$ increases when the fluid flow rate, i.e., fluid inertia, increases or the fluid kinematic viscosity decreases (Equation (3)). This force imbalance produces a fluid velocity gradient in the flow. Eventually the $R_{e}$ imbalance becomes too large, producing fluid eddies and vortexes associated with turbulent fluid flow. The transition from laminar flow to turbulent flow is not a sharp transition. A fluid stream can often contain regions of laminar, chaotic advection, and turbulent flow. $R_{e}$ < 1000 flows are primarily laminar. An $R_{e}$ > 2000 is typically considered a primarily turbulent flow, while an $R_{e}$ > 5000 is usually a completely turbulent flow. The small channel dimensions in microfluidic mixers operate at low fluid flow rates. So, turbulent flow rarely occurs in true microfluid-mixing devices.

What is the $l_{st}$ produced by turbulent mixing? The turbulent fluid-mixing process can be described as the kinetic energy transfer process or cascade within the fluid [35]. The fluid motion transition from laminar flow into a turbulent flow occurs when the laminar flow kinetic energy is transferred to the kinetic energy of fluid eddies and vortexes. This process has been described as an energy mixing “cascade”. The laminar flow fluid kinetic energy is initially transferred to large vortical structures or eddies. The kinetic energy of these large eddies is then transferred to smaller and then smaller eddies. Finally, when the fluid eddies are small enough, the fluid viscosity starts to convert the kinetic energy of the smallest eddies into thermal energy [35]. At this point, the $l_{st}$ distance between the small fluid eddies will be described by the Kolmogorov Length, $l_{K}$ (Equation (9)).

(9) lK=ν3ε14

where

$l_{K}$ = Kolmogorov Length (m);
ν = kinematic viscosity (m2s);
ε= energy dissipation rate (Js∗kg).

**Figure 4:**
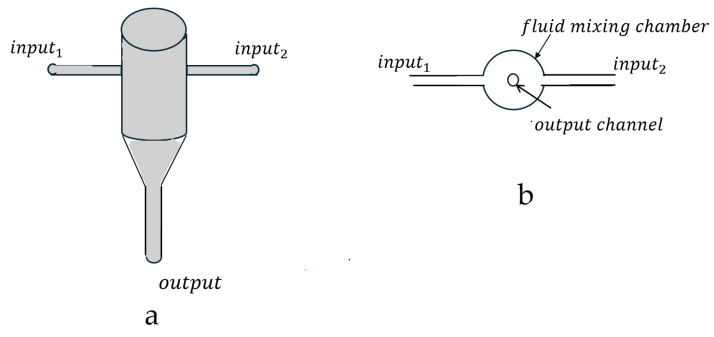
Simple drawings of a Confined Impinging Jet (CIJ) turbulent flow mixer, showing the side view (**a**) and top view (**b**) of the turbulent mixer.

Johnson and Prud’homme constructed a lab-scale Confined Impinging Jet (CIJ) mixer where two impinging jets of fluid collided in a confined mixing chamber. The authors then quantitatively evaluated the small-scale CIJ turbulent “micromixing” process [37]. A simple diagram of the CIJ turbulent mixer is shown in Figure 4.

A simple low pressure laboratory scale syringe pump system was sufficient to push the two impinging fluid streams at fluid flow rates necessary to produce a turbulent micromixed state. A rapid competitive chemical reaction ruler reaction was used to determine the absolute mixing performance of this CIJ mixer [38]. The goal of this study was to study the mixer characteristic, not to formulate mRNA LNPs. An aqueous stream of dimethyloxypropane (DMP) with dissolved NaOH base was injected into the mixing chamber through port A. Another aqueous stream of HCl acid was injected through port B. The DMP will be hydrolyzed only if the acid in the B stream can neutralize the hydroxide in the A stream. DMP hydrolysis will release two methanol molecules, which can be measured from the output at port C. The acid–base neutralization reaction does not control the DMP reaction time, because it is always much faster than the fluid-mixing time. The DMP hydrolyzation to methanol is controlled by the micromixing time, $t_{m}$, of two CIJ input streams. DMP output concentration depends on fluid mixing and can be used to determine the $t_{m}$ of the CIJ mixer. The DMP concentration in the output stream was measured by gas chromatography (GC) [37].

Baldyga and Bourne [39] previously theoretically calculated the micromixing time, $t_{m}$, proportionality for static turbulent mixer designs similar to the CIJ device used in Johnson and Prud’homme study [37,39,40] (Equation (10)).

(10) tm ∝  14 νϵ12

The energy dissipation rate, ε, is the energy rate input into the CIJ mixing volume, and it is given by Equation (11).

(11) $$\varepsilon =\frac{P}{\rho V_{m}}$$

where

*P* = input energy (Js);
ρ = fluid density ($Kg/m^{3}$);
$V_{m}=$ mixing volume ($m^{3}$).

The input mixing power, P, is the kinetic energy per unit time of the colliding fluid streams in a CIJ turbulent mixer. Considering the simplest CIJ case where the two impinging jet fluid streams have identical kinetic energy, we have $P\;\propto mu^{2}$, where m is the mass-flow rate ($\frac{Kg}{s}$), and u is fluid flow ($\frac{m}{s}$) of the two colliding fluid streams. Combining Equations (11) and (12), together with the dimensions of the CIJ turbulent mixer, give the $t_{m}$ proportionality equation (Equation (12)) [37]:

(12) τm ∝ 122 ν12∆32 d12u32

where

u= fluid flow rate (ms);
z = distance between the two CIJ fluid injection ports (m);
d = fluid injection stream diameter at the two input injection ports (m).

Δ=zd

Equation (3) indicates that the fluid flow, $R_{e}$, is proportional to the flow rate u. Equation (13) predicts the turbulent mixing τm ∝ Re−32. Input stream fluid $R_{e}$ was varied from 10 to 3820 in this CIJ mixing study. The experimental chemical ruler results indicated that micromixing time, $t_{m}$, for all turbulent flow conditions were proportional to u−32. This is consistent with the proportionality Equation (13). This important study quantitatively characterized the mixing performance, i.e., $t_{m}$, of a fluid flow CIJ mixer a high $R_{e}$. This quantitative analysis can be used to estimate and control CIJ micromixing performance and scale-up for clinical supplies and manufacturing [37].

**Figure 5:**
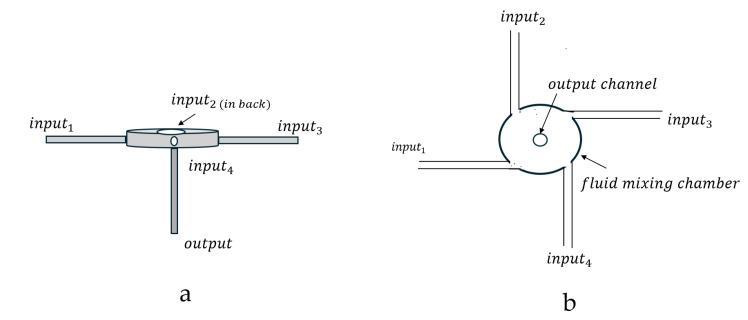
Simple drawings of 4-jet multi-inlet vortex mixer (MIVM), showing side view (**a**) and top (**b**) of the turbulent mixer.

In 2008, Ying Liu [41] et al. introduced the muti-inlet vortex mixer (MIVM) design for formulation of biologic and vaccine nanoparticles. This MIVM had a four-input stream design. The four MIVM fluid input streams tangentially enter the mixing chamber at turbulent fluid flow velocities, u. The mixed fluid flows then exit the mixing chamber through a center output port as schematically illustrated in Figure 5.

The MIVM is a turbulent mixer designed to overcome some of the limitations of turbulent CIJ mixers. The MIVM retains rapid turbulent mixing but is more flexible and easier to operate and scale up [41,42]. The solvent (organic solvent) and anti-solvent (aqueous) fluid streams that collide in a CIJ mixer must have equal and opposite momentum to prevent fluid “back flow” in one input stream. This often limits the volumetric mass flow ratio of the anti-solvent to solvent fluid streams to around 1.0 for a CIJ mixer. This produces a CIJ output fluid which is approximately 50% of solvent fluid by volume. This high concentration of the solvent, e.g., ethanol, will typically need to be removed in subsequent process steps such as dialysis or tangential flow filtration (TFF). In contrast, MIVM turbulent mixers do not require an equal momentum fluid stream collision like CIJ mixer to prevent backflow problems. The MIVM input fluid streams do not directly collide. They meet in the center of the MIVM mixer and then exit through the center output port. Many fluid flow arrangements are possible, such as one or more input fluid streams, which can have a high volumetric flow rate, while other fluid input fluid streams could have a lower volumetric flow rate. Turbulent mixing would still occur in the center of the MIVM. This allows for the operational advantage of mixing conditions where, if necessary, the final solvent to anti-solvent flow rate ratio range can be varied over a wider range. This makes the solvent removal process after nanoparticle mixing easier, since less solvent can be introduced to the final fluid output. Having four separate fluid input streams also enables the introduction of multiple pharmaceutical molecules into the self-assembly process [42,43].

The primary purpose of Ying Liu et al.’s [41] study was to characterize the mixing performance of the MIVM device using the acid–base DMP chemical “ruler” reaction described in Johnson and Prud’homme [37]. DMP hydrolysis was again used as a surrogate for MIVM mixing efficiency. The composite $R_{e}$ for a MIVM mixing device is the sum of the $R_{e}$ of the individual fluid entry ports [41] (Equation (13)).

(13) $$R_{e}=\sum_{i=1,\;4}\frac{\mu_{i}}{\nu_{i}}\;d\;$$

where

$\mu_{i}$ = fluid velocity at input i, (ms);
$\nu_{i}$ = kinematic viscosity at input i (m2s);
d = mixing chamber diameter (m).

The MIVM device mixing efficiency was determined over a $R_{e}$ range from 50 to 5000. Suitable turbulent mixing conditions were obtained for $R_{e}$ > 1600 with various combinations of fluid input arrangements. These experiment results were compared to computational fluid dynamics (CFD) simulations of the fluid mechanics and chemical reactions in the MIVM [41]. An excellent correlation between the experimental mixing results and the CFD simulations were found for $R_{e}$ > 800. A smaller scale µMIVM was developed which accurately scaled to the previously developed larger MIVM [43]. This lab-scale µMIVM requires only small amounts of experiment reagent to operate successfully. This is a significant advantage for early development formulation screening studies.

## 5. Flash Nanoprecipitation, Nanocomplexation, and mRNA Incorporation

Once rapid mixing of the fluid is complete how does the process of nanoparticles self-assembly proceed? D’addio and Prud’homme [44] have described the flash nanoprecipitation process for turbulent mixers as a rapid precipitation of hydrophobic molecules dissolved in an organic solvent when mixed into an excess hydrophilic anti-solvent. Hydrophobic molecules, e.g., lipids or water-insoluble drugs, will dissolve in organic solvent, e.g., ethanol. The hydrophobic molecules will rapidly precipitate into nanometer sized particles when rapidly mixed with an excess of a hydrophilic anti-solvent like water or saline. This process has been referred to as “flash” nanoprecipitation (FNP) by solvent-exchange [37,45]. The Kelvin equations [46] can be used to model this nanoparticle nucleation and growth as a phase separation into spherical particles that reduces the free energy of the system. In this model of nanoparticle formation, the solution supersaturation of the hydrophobic molecules has the most significant influence on FNP particle formation and final diameter. The supersaturation value of a typical hydrophobic pharmaceutical molecule is generally defined in Equation (14) [44].

(14) $$S\;\equiv \frac{c_{s}}{c_{\infty }}$$

The $c_{s}$ value equals the concentration of the hydrophobic molecule at or near the nanoparticle surface. The initial $c_{s}$ concentration is the total amount of hydrophobic molecules dissolved in hydrophobic solvent divided by the final mixed solvent and anti-solvent solution volume just as the nanoparticles begin to form. The maximum solubility concentration of the hydrophobic molecule in the anti-solvent/solvent solution at a great distance from the nanoparticle is considered $c_{\infty }$, which is the highest possible concentration of non-absorbed hydrophobic molecules in the aqueous anti-solvent. In general, this is just the maximum solubility of the hydrophobic molecules in the anti-solvent. So, the supersaturation, S, value compares how much of the hydrophobic agent was added to mixed total volume to the solubility of non-absorbed hydrophobic molecules in the anti-solvent, e.g., water or saline. The S value will be high when a significant amount of anti-solvent dissolved hydrophobic molecules are quickly transferred from the anti-solvent to nanoparticle surface. A molecule in the solvent fluid that can remain more dissolved in the anti-solvent fluid, rather than absorbing nanoparticles, will have a relatively low S value. The critical equilibrium FNP radius, $r_{c}$, at a supersaturation value, S, is determined by the Kelvin equations (Equations (15) and (16)) [44].

(15) $$ln\frac{c_{s}}{c_{\infty \;}}=\frac{2\gamma M}{\rho RTr_{c}}=ln\;S$$

(16) $$r_{c}=\frac{2\gamma M}{\rho RT(lnS)}$$

where

γ = particle surface tension;
M = hydrophobic molecule molecular weight;
ρ = density hydrophobic molecules;
R = gas constant;
T = temperature.

These simple Kelvin equations predict that the average nanoparticle diameter (${2r}_{c})$ for a rapid well-mixed solvent-exchange process should decrease with lnS and T. This flash nanoprecipitation analysis applies equally well to a well-mixed microfluidic process because this is also a solvent-exchange process. Eventually, however, all the available “completely dissolved” hydrophobic molecules in the mixed solution will be incorporated into the growing number of FNP nanoparticles. At this point, the concentration of the hydrophobic molecules near the nanoparticle surface, $c_{s}$, will then approach $c_{\infty }$. The $c_{\infty }$ concentration could have almost any value; however, when $c_{s}\approx c_{\infty }$, the supersaturation is gone. At this point, nanoparticle diameter growth will slow down and eventually stop. This process is what happens when a hydrophobic molecule which is initially significantly above the solvent critical micelle concentration (CMC) rapidly precipitates out of the solution. Lipid FNP nucleation and growth is certainly a major process for mRNA LNP formation, and it is certainly a more complex process than this very useful simplification. However, lipid FNP is clearly not the only process involved with complete mRNA LNP formation.

The rapid turbulent solvent-exchange procedure using large solubility differences is not the only path to self-assembling nanoparticles. Nanoparticle “complexes” can also rapidly self-assemble after fluid mixing as the result of other attractive molecular forces, such as molecular electrostatic and/or non-covalent interactions. Hu et al. [42] have referred to this process as flash nanocomplexation (FNC). In general, the FNC process does not rely solely on rapid hydrophobic-to-hydrophilic solvent-exchange mechanisms to promote nanoparticle formation. There is strong evidence that the FNC process is involved in standard mRNA LNP formulation processes [29].

The molecular details of the mRNA LNP self-assembly process have been the focus of much biochemical and biophysical research [18,47,48,49,50]. Self-assembly of mRNA LNP vaccines via either microfluidic mixing or turbulent fast nanoprecipitation (FNP) is clearly a complicated multi-step process. The first step is to combine “solvent” ethanol stream containing four distinct lipid-soluble components, namely (1) an ionizable cationic lipid, (2) cholesterol, (3) pegylated lipid, and (4) bilayer-forming “helper” phospholipids, with the “anti-solvent” aqueous stream containing mRNA with a pH 4 buffer [51]. The standard ionizable cationic lipids, e.g., Dlin-KC2-DMA (2,2 dilinoleyl-4-(2-dimethylaminoethyl)-1,3-dioxolane) (Dlin—KC2-DMA) and MC3 (Dlin-MC3-DMA), will be positively charged at pH 4.0. The positively charged lipid will “complex” with the negatively charged mRNA to form an electrostatically close-to-neutral core particle. This mRNA–cationic lipid core is then coated with the other lipid molecules that were dissolved in the ethanol. This lipid coating process continues until sufficient pegylated lipid, e.g., DMG-PEG2000 (1,2-dimyristoyl-rac-glycero-3-methoxypolyethylene glycol-2000) has coated the mRNA–cationic core to inhibit additional nanoparticle growth and aggregation. So, mRNA LNP self-assembly by either microfluids or turbulent mixing involves at least two self-assembly processes [29]: (1) the lipid assembly driven by hydrophobic attraction due to the solvent-exchange process, i.e., FNP [44]; and (2) the mRNA cationic lipid complex driven by electrostatic attraction, i.e., FNC [42]. The pH is then raised to above pH 7.0 during dialysis or TFF to start further rearrangements in the structure of mRNA LNP solution.

This interpretation of mRNA LNP is supported by a recent investigation that used multi-wavelength fluorescence spectroscopy to quantitatively tag and measure to the mRNA and “helper” lipid content of single LNP particles during the formulation process [29]. A simple T-mixing device was used to mix an ethanol stream containing the LNP lipids with an aqueous stream of mRNA at approximately 30% *v*/*v* ethanol at pH 4.0. A simple $R_{e}$ calculation using the T-mixer, input tubing, and fluid flow rates results in $R_{e}$ ≈ 1900. This suggests that significant turbulent mixing occurred in this T-mixing system and that the two input fluid streams were rapidly mixed. Following this turbulent mixing step, the ethanol–aqueous LNP solution was dialyzed against a pH 7.4 aqueous buffer to eliminate the ethanol and complete the mRNA LNP formulation process. Fluorescence multi-laser cylindrical illumination confocal spectroscopy (CICS) of single nanoparticles revealed several distinct populations of nanoparticles with different mRNA “payloads” at pH 4.0 before the ethanol removal dialysis. Three populations of nanoparticles were observed at ≈ 30% *v*/*v* ethanol pH 4.0 prior to ethanol removal via dialysis:

(1) Lipophilic complexes which contained some mRNA;
(2) Positively charged lipophilic particles with no mRNA (empty LNPs);
(3) Non-lipophilic complexes which contained mRNA.

**Figure 6:**
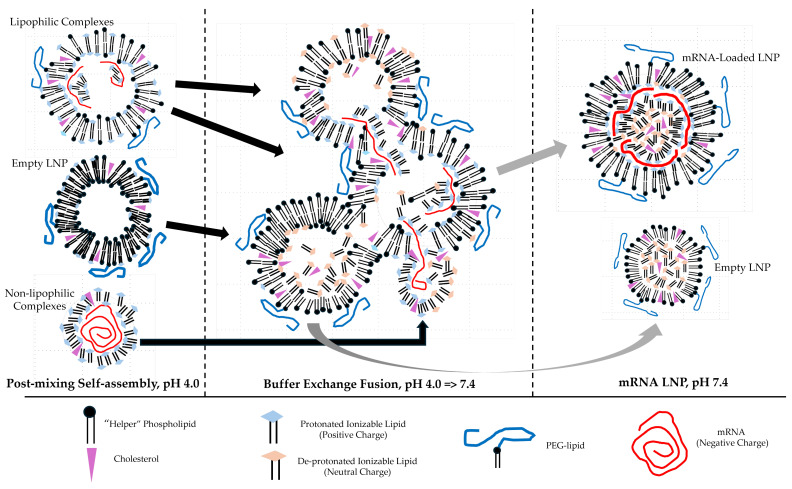
Simplified mRNA-LNP post-mixing particle fusion proposal for forming mRNA LNP. From Li et al., “Payload distribution and capacity of mRNA lipid nanoparticles [29].

The combined average ζ-potential of all these particles measured by electrophoretic light scattering (ELS) was +45 ± 1 mV. In contrast, different LNP populations with different mRNA payloads were observed after ethanol removal via dialysis at pH 7.4. A significant population of mRNA-loaded mRNA LNP particles were observed together with a large population of “empty” LNPs at pH 7.4. The combined average ELS ζ-potential of these post-dialysis nanoparticles was −6.3 ± 1 mV. The N/P ratios and percentage of pegylated lipid in the formulations were varied to better understand this complicated kinetically controlled self-assembly mechanism. The authors speculate that these LNP population changes are occurring early during the dialysis ethanol-removal process, when the ethanol concentration is still relatively high. Some of the author’s surprising results are presented in Table 1 and Figure 6. Previous observations have shown that ethanol-induced liposome fusion at 30 to 40% *v*/*v* ethanol with siRNA cationic ionizable lipid particles at pH 7.4 encapsulates high levels of siRNA [52,53].

In addition to mRNA LNP formulation, MIVM and CIJ turbulent mixing devices have also been shown be highly efficient at preparing nanoparticles of hydrophobic peptides, biologics, and small-molecule drugs for pharmaceutical applications [54]. Recently, the turbulent FNP mixing process has been extended to include nanoparticle encapsulation of hydrophilic and charged pharmaceutical molecules using inverse FNP (iFNP) and hydrophilic ion pairing [55,56]. For example, the hydrophobic anti-malaria drug Lumefantrine (LMN) was incorporated into 200 nm hydroxypropyl methylcellulose acetate succinate (HPMCAS) nanoparticles by FNP, using (1) a small lab-scale CIJ, (2) a development-scale MIVM, and (3) a clinical production-scale MIVM mixing device [57]. When scaled to the same $R_{e}$ each mixing device produced indistinguishable nanoparticles with the same particle diameter and polydispersity. This study was a clear demonstration of the easy scalability of FNP technology. More mathematical insight into CIJ and MIVM FNP mixing dynamics is provided by several CFD studies [58,59].

## 6. Formulation of Empty and mRNA LNPs with Microfluidic Mixers

The potential advantages of microfluid chaotic advection mixing to prepare nanoparticles of all types have been well known for over 20 years [23,24,60]. Maeki et al. [61] carefully examined the formation mechanism of empty 1,2-dioleoyl-sn-glycero-3-phosphocholine (DOPC) lipid nanoparticles without mRNA, i.e., “empty” LNP, by chaotic microfluidic mixers. These authors investigated the microfluidic mixing total flow rate (TFR) and flow rate ratio (FRR), which are two Critical Process Parameters (CCPs) that influence LNP particle diameter. They manufactured three microfluidic SHM devices that were used to mix an ethanol stream $\left({fl}_{eth}\right)$ containing the phospholipid DOPC with a saline stream (${fl}_{aqu}).$ Mixing experiments were performed with different ${fl}_{aqu}$/${fl}_{eth}$ values, i.e., FRRs, and different ${fl}_{aqu}$ $+\;{fl}_{eth}$ values, i.e., TFRs. Two of the mixers had different herringbone structure sizes (11 and 31 µm) to create different chaotic advection mixing conditions while the channel of the third microfluidic mixer was clear of obstructions. The ${fl}_{eth}$ stream was also labeled with a rhodamine phospholipid to measure the rhodamine distribution in the mixing channel with a scanning laser confocal fluorescence microscope. This allowed the authors to quantitatively measure the mixing of the ${fl}_{eth}$ and ${fl}_{aqu}$ fluid streams as they traveled along the mixing channel. The resultant POPC LNP average diameter and polydispersity index (PDI) of the output stream were measured by dynamic light scattering (DLS) for the three SHM devices. The resultant POPC LNP DLS diameters ranged from 52 to 118 nm for FRR = 3, and 32 to 48 nm for FRR = 9, depending on the TFR and the channel diameter of the herringbone mixer. The resultant POPC LNP diameter depended on a combination of the microfluidic mixing input parameters. The authors observed the following: (1) when the TFR is held constant, the LNP diameter decreases with the increasing FRR; and (2) when the FRR is held constant, the LNP diameter decreases with the increasing TFR. The largest effect was the dimension of the SHM herringbone obstructions. The larger 31 µm herringbone obstructions produced small-diameter LNPs, ranging from 32 to 61 nm, compared to the 11 µm herringbone obstructions, which produced LNP diameters from 38 to 118 nm. Fluorescence confocal mixing measurements along the mixing channel clearly indicated where the ${fl}_{eth}$ and ${fl}_{aqu}$ fluid stream mixed. It is clear from the fluorescence imaging results that input mixing parameters which produce with faster mixing times also produce smaller LNP diameters. The authors estimated the ethanol concentration in the mixing channel during the formulation of the POPC LNPs. The POPC LNP formation occurred at a critical ethanol concentration of 60 to 80%. LNPs were also stable at or below this critical concentration range, so the final ethanol concentration after mixing was not reduced after the microfluid mixing process. The authors propose a POPC empty LNP formulation mechanism during microfluidic mixing which involves the formation of semi-stable bilayer phospholipid fragments (BPFs) that are intermediate [62].

A more comprehensive study of scalable microfluid manufacturing of liposome drug delivery nanoparticles was performed by Webb et al. [63]. These authors compared microfluidic encapsulation of ovalbumin protein (OVA) using either an SHM or TRM microfluidic mixer. The effect of different liposome chemical compositions on final nanoparticle diameter, polydispersity index (PDI), and OVA encapsulation efficiency was also examined. Issues involved in prepared mixed pharmaceutical-grade nanoparticles such as reducing the final-product ethanol concentration and concentrating the final nanoparticle product by tangential flow filtration were evaluated.

Ripoll et al. [30] examined the microfluidic mixing input CCP for the four-ring Cytiva NxGen (Cytiva, Marlborough, MA, USA) microfluidic TRM device. TRM devices are capable of higher fluid flow rates than SHM devices. Curved TRM devices also have the advantage of inertial (Dean) vortices below the turbulence onset, thus improving mixing efficiency. This makes TRM devices better suited for microfluidic-device scale-up to manufacturing. The fluid flow conditions in the TRM channel were measured by adding fluorescence dye to the ${fl}_{eth}$ stream. The microfluidic mixing in the device channel was quantified by the cross-section fluorescence intensity using a fluorescence microscope and computer image analysis. TRM fluid mixing was “poor” at FRR = 3 for TFR < 0.4 mL/min ($R_{e}=22$). “Highly mixed” microchannel conditions required TFR > 4 mL/min ($R_{e}=220$) at FRR =3. A complete turbulent fluid flow ($R_{e}>2000$) was not obtained at either TFR rate. This TRM device was used to prepare pDNA LNPs. The LNP lipids used in this pDNA encapsulation study were the cationic lipid Dlin-MC3-DMA (MC3), DOPC, cholesterol, and 1,2-dimyristoyl-rac-glycero-3-methoxypolyethylene glycol-2000 (DMG-PEG2000) at a 50:10:38.5:1.4 mole ratio.

Acceptable mRNA LNP critical quality attributes (CQAs) are shown below:

(1) Z-average intensity DLS diameter near 100 nm;
(2) DLS polydispersity index (PDI) < 0.2;
(3) LNP nucleic acid encapsulation efficiency (EE) > 80%;
(4) Consistent cryo-electron microscopy (Cryo-EM) structures;
(5) Average LNP ζ-potential between 0 and −5 mV.

The microfluid TRM device used by Ripoll et al. [30] easily achieved the first four of these CQAs at TFR > 4 mL/min and FRR > 3. The LNP ζ-potentials were not measured in this study. LNP ζ-potentials are typically measured using electrophoretic light scattering (ELS) or, more recently, by capillary isoelectric focusing (CEI) technology [64]. In addition, the CPP necessary for the pDNA LNP CQAs described above correlated with TFR and FRR inputs necessary for “highly mixed” fluids. A more detailed analysis of LNP microfluid manufacturing considerations can be found in Roces et al. [65]. The general rule from this study was that increasing TFR and FRR will decrease nucleic acid LNP particle diameter. Recent Design-of-Experiment (DOE) studies have been performed to assess the effect of microfluidic mixing input CPP on the lab-scale preparation of siRNA LNPs [66] and self-amplifying mRNA (saRNA) LNPs [67].

Biodegradable ionizable cationic lipids were introduced into LNPs to deliver siRNA in 2013 by Maier et al. [68]. Pardi et al. [69] reported that cationic liposomes containing associated nucleoside-modified mRNA could transfect both cells and mice with luciferase mRNA. These mRNA LNP particles were formulated using simple microfluid T-junction assembly with standard HPLC tubing to mix an ${fl}_{eth}\;$ of lipid with an ${fl}_{aqu}$ of mRNA [68]. Significant levels of luciferase protein were produced in mice for 1 to 4 days after injection, demonstrating the potential of mRNA transfection. Also, in 2015, Leung et al. [51] used the ionizable cationic lipid, 2,2 dilinoleyl-4-(2-dimethylaminoethyl)-1,3-dioxolane (DLin—KC2-DMA), DSPC, cholesterol, and PEG-lipid to prepare LNPs using a staggered herringbone mixer (SHM) microfluidic device. Anionic biopolymers siRNA, mRNA (1.7 kb), pDNA (6 kb), and negatively charged gold nanoparticles were all incorporated into SHM-prepared LNPs [51,70,71,72,73]. Cryo-EM images of these siRNA particles were 50 to 100 nm in diameter, with an electron-dense particle interior [51,70]. The siRNA LNP encapsulation efficiency of siRNA was as high as 90% with the right LNP lipid composition. This SHM microfluid mixer design would be become the basis for the NanoAssemblr™ Benchtop™ mixer which was later marketed by PrecisionNano Systems LLC (Vancouver British, Columbia, SC, Canada) [51,74].

Yanez Arteta et al. [75] compared the biophysical structure to the in vitro mRNA uptake and protein expression using the NanoAssemblr Benchtop microfluid mixer in 2018. DLS particle sizing, Cryo-TEM, and small-angle X-ray scattering (SAXS) were used to characterize the size and structure of the mRNA LNPs. This mRNA LNP structural information was compared with human erythropoietin (hEPO) mRNA uptake and protein production in two cell lines. Both the LNP diameter and surface structure were found to be critical for high mRNA protein expression. The mRNA LNP surface structure was believed to be important for mRNA endosomal escape to the cytoplasm. Another biophysical stability study of COVD-19 mRNA LNPs indicated an mRNA LNP structure with an ionizable cationic lipid/mRNA core surrounded by an outer coat of 1,2-distearoyl-sn-glycero-3-phosphocholine (DSPC)/cholesterol “helper” lipids [76]. Hydrolysis of mRNA in the LNP core appeared to be a major contributor to mRNA-LNP instability [76]. The cationic lipid/mRNA LNP core was also examined with the mRNA binding dye thionine to enhance the contrast of mRNA in cryo-EM images of mRNA LNP [77]. The mRNA thionine-“stained” image assay suggests intriguing mRNA structures within the LNP core.

Li et al. [29] have used fluorescence multi-laser cylindrical illumination confocal spectroscopy (CICS) to detect and characterize fluorescently labeled mRNA LNPs, empty LNPs, and free mRNA at the single-nanoparticle level. This allows the CICS instrument to differentiate between the following individual particles in the mRNA LNP formulation:

(1) mRNA-loaded LNP;
(2) Empty LNP;
(3) Free mRNA nanoparticles.

This very powerful single-nanoparticle characterization technology was used to determine the percentages of mRNA LNPs, empty LNPs, and free non-encapsulated mRNA. The amount of mRNA in an individual mRNA LNPs could also be estimated. Selected results of this study are presented in Table 2 and Figure 6.

How do mRNA LNPs’ basic biophysical parameters, which are discussed above, influence in vitro cell-based potency? Tong [79] et al. have correlated the several mRNA LNP biophysical properties with an in vitro cell-based potency. The mRNA LNP formulations used in this study were prepared using a microfluidic T-junction device [80]. The in vitro cell-based assay is described in [81]. The mRNA LNP transfection potency in HepG cells was monitored as a function of mRNA LNP dose, storage conditions, and storage time. The in vitro mRNA LNP potency did not significantly change until the LNP particle size was larger than 130 nm in diameter. However, there was rapid mRNA degradation and potency loss over the course of several days at temperatures above ≥25 °C. However, the percentage of mRNA encapsulated in the LNPs did not change significantly with storage conditions. Cell-based mRNA LNP transfection assays can provide valuable insight into the cellular mechanism of mRNA LNP uptake and protein translation. However, cell-based mRNA LNP potency transfection assay results may not correlate with in vivo efficacy.

The rapid development of COVID-19 mRNA LNP vaccines by Pfizer Inc. (New York City, NY USA) and Moderna Inc. (Cambridge, MA, USA) was a spectacular achievement of 21st-century medical science [82]. Even before the start of the COVID-19 pandemic, both Pardi et al. [83] and Liang et al. [84] demonstrated that mRNA LNP vaccines could elicit anti-hemagglutinin influenza antibodies in mice and Rhesus Macaques, respectively. In addition, Richner et al., [85] demonstrated that an mRNA LNP vaccine was effective an Zika virus vaccine in mice. Bahl et al. [86] also demonstrated the effectiveness of an influenza mRNA LNP vaccine in humans in a 2017 publication. This critical pre-pandemic mRNA LNP influenza research was then rapidly applied to successfully preparing COVID-19 mRNA LNP vaccines [87,88,89]. These results and other microfluidic mixing mRNA LNP formulation structure-to-function relationships are discussed in two excellent reviews [71,90,91,92].

## 7. Formulation of mRNA by Turbulent Mixing

Most lab-scale nucleic acid nanoparticle formulation studies currently are lipid formulations performed with microfluidic technology [51,69,70,71]. This is certainly the situation because of (1) the desire for small-scale formulation using minimal amounts of expensive nucleic acid material, and (2) the commercial availability of easy-to-use microfluid mixers microfluidic mixers [28]. The commercial scale formulation of mRNA LNP vaccines was not a major research concern until the COVID-19 pandemic in 2020. A solvent-exchange precipitation process to scale up self-assembled siRNA LNPs for clinical trials was reported in 2014 [93]. This siRNA LNP formulation was optimized to significantly reduce Ostwald ripening of the LNPs [94]. This process was later used to prepare an RSV mRNA LNP vaccine for pre-clinical studies [95]. Pre-clinical and clinical mRNA LNP vaccine studies had demonstrated general safety and efficacy prior to the COVID-19 pandemic [83,84,86].

The rapid, reproducible formulation of large amounts of mRNA LNP vaccine soon became a major concern during 2020. At the start of the pandemic in 2020, the two mRNA LNP formulation mixing schemes were under consideration for manufacturing scale-up. One scale-up approach was a microfluidic “scale-out” by fabricating a 128 parallel channel SHM chaotic advective microfluid mixer in a single mixing device [96]. Pfizer Inc. ultimately decided to use the FNP approach to manufacture Comirnaty™ (Pfizer, New York, NY, USA) with a specially designed CIJ mixer [97]. The CIJ mixer design was probably chosen by Pfizer Inc. because of reproducible results, low fouling, and high throughput [98]. Pfizer Inc. most likely “scaled up” Comirnaty™ production with multiple CIJ mixers in parallel to increase product throughput. Unfortunately, the details of CIJ mixing process used to manufacture Comirnaty™ are not publicly available. Practical information about lab-scale CIJ and MIVM formulation of mRNA LNP vaccines has been recently published by Subraveti et. al. [78]. Formulation-solution recipes and detailed step-by-step instructions on how to prepare lab-scale mRNA vaccines by CIJ or MIVM devices are provided. The ethanol solvent was removed by dialysis in a pH 7.4 HEPES buffer after the mRNA LNPs were formed for both processes. The mRNA LNPs prepared with the CIJ or MIVM had similar biophysical properties and HeLa cell in vitro transfection properties. In contrast, the mRNA LNPs prepared by manual pipette mixing of the solvent (lipids) and anti-solvent (mRNA) were biophysically different from CIJ and MIV prepared mRNA LNPs. In addition, manually pipetted mRNA LNPs had a significantly lower ability to transfect HeLa cells. The post-dialysis mRNA LNP biophysical properties for this study are summarized in Table 2.

In addition to mRNA LNP formulation, MIVM and CIJ turbulent mixing devices have also been shown be highly efficient at preparing nanoparticles of hydrophobic peptides, biologics, and small-molecule drugs for pharmaceutical applications [54]. Recently, the turbulent FNP mixing process has been extended to include nanoparticle encapsulation of hydrophilic and charged pharmaceutical molecules using inverse FNP (iFNP) and hydrophilic ion pairing [55,56]. For example, the hydrophobic anti-malaria drug Lumefantrine (LMN) was incorporated in to 200 nm hydroxypropyl methylcellulose acetate succinate (HPMCAS) nanoparticles by FNP using (1) a small lab-scale CIJ, (2) a development-scale MIVM, and (3) a clinical production-scale MIVM mixing device [57]. When scaled to the same $R_{e}$ each mixing device produced indistinguishable nanoparticles with the same particle diameter and polydispersity. This study was a clear demonstration of the easy scalability of FNP technology. More mathematical insight into CIJ and MIVM FNP mixing dynamics is provided by several CFD studies [58,59].

## 8. Micromixing Practical Information and Future Directions

Microfluidic or turbulent are two basic mixing strategies for preparing for mRNA LNPs. A process-robustness comparison between two microfluid and one turbulent process was reported by O’Brien Laramy et al. [48] for both siRNA and mRNA. A standard LNP solvent-exchange formulation method was used by mixing a fluid stream of lipid dissolved in ethanol with an aqueous stream of dissolved mRNA or siRNA. The authors performed a three-level full-factorial DOE for each mixing process using the following input variables:

(1) Flow rate ratio (FRR);
(2) Ratio of ionizable amines to nucleic acid phosphates, i.e., the N/P ratio;
(3) Ionizable lipid (MC3) formulation mole percentage.

The two microfluidic mixers were SHM and TRM designs [28]. These microfluidic mixers were operated at a $R_{e}$ < 100. The turbulent mixer was a jet co-flow mixer from DIANT Pharma, Inc. which operated at 1000 < $R_{e}$ < 2000. The LNP diameter, LNP PDI, RNA efficiency %, and the SAXS value $q_{0}$ peak position after a phosphate buffer dialysis procedure were the DOE outputs. CryoEM was also used to compare the siLNP samples. Examination of the DOE output reaction spaces indicated that the siLNPs prepared using turbulent flow mixer had significantly smaller average particle diameters and narrower particle size distributions than the microfluidic mixing devices. There was no significant difference in the RNA encapsulation efficiency between the two basic mixing technologies. Unfortunately, in vitro or in vivo studies were not performed with siRNA LNP formulations to determine if there was a difference in biological activity.

Table 3 summarizes the pharmaceutic conditions used to prepare a selection of nucleic acid delivery LNP formulations.

Another RNA LNP DOE study used 10 input factors for different self-amplifying RNA (saRNA) LNP formulations [67]. A turbulent T-junction mixing process was used in study. The study optimized the saRNA LNP formulations for following five final formulation critical quality attributes (CQAs):

(1) LNP particle diameter;
(2) LNP encapsulation efficiency;
(3) RNA integrity;
(4) In vitro protein expression;
(5) In vitro IL-6 release [67].

The study provided insights into the LNP encapsulation of higher MW RNA molecules like saRNA or multigene expression systems [67]. These recent DOE studies provide valuable insight into the complex process of RNA LNP formulation. In the future, artificial intelligence and machine learning (AL/ML) analysis of larger datasets will be certainly used to direct mRNA LNP formulation development [99,100].

What are the relative advantages of the two mixing processes for the development, scale-up, and manufacture of mRNA? Formulation development of an mRNA LNP vaccine requires the evaluation, i.e., screening of many formulation compositions for (1) biophysical properties, (2) in vivo biological activity, and (3) pre-clinical safety and efficacy studies. Minimal use of expensive reagents, e.g., mRNA and ionizable lipids are therefore a critical cost consideration during early bench-scale formulation development. The low fluid flow rate and $R_{e}$ of small-scale chaotic advection microfluidic mixing is well suited for mRNA LNP formulation at a 1 to 3 mL scale. This minimizes the use of critical reagents. Thus, microfluidic mixing technology is better suited to prepare lab-scale mRNA LNP batches for developmental research [65,101]. However, screening a large number mRNA LNP formulations with a single microfluidic mixer is a tedious and time-consuming process. Automation to efficiently prepare numerous mRNA LNP for high throughput (HT) formulation screening has been a high priority, but a major resource and technical challenge for most research labs [102,103,104]. Fortunately, formulation HT screening chaotic advection microfluid mixing technologies are now commercially available. The NanoGenerator Flex M&M Premium manufactured by PreciGenome LLC (San Jose, CA USA) and the Sunscreen (HT) by Unchained Labs (Pleasanton, CA, USA) do have automated high throughput muti-formulation capabilities [105,106]. A table of commercially available microfluidic and turbulent flow mixers are provided in Table 4a and 4b respectively.

Lab-scale chaotic advection microfluidic mixing devices are difficult to scale up to manufacturing or even early clinical study-scale production [107]. The low fluid flow rates, i.e., $R_{e}$, of chaotic advection fluid devices result in an LNP formulation product throughput that is too low for cost-effective manufacturing [78,97,107]. The high surface contact between the fluid and the walls of the microchannel certainly contributes to the clogging problems frequently observed when microfluidic mixers are operated at high flow rates [57,78,107]. Increasing the flow rate in microchannel devices is typically not practical due to (1) high shear and (2) microfluidic device clogging in small microfluidic channels. Designing chaotic advection mixing devices with multiple parallel microfluidic mixers is challenging [96]. It is likely that a highly parallel microfluid-mixing device would add mixer design complexity without solving the microfluid channel-clogging problems.

Turbulent flow mixing, of course, is the alternative mixing process to microfluidic chaotic advection and T-mixing. Turbulent mixing typically requires an $R_{e}$ > 2000 for highly turbulent mixing times on a sub-millisecond timescale. According to Equation (3), a high $R_{e}$ requires a relatively high fluid velocity (μ) and large “characteristic length” (l). Typical CIJ or MIVM turbulent mixing devices can efficiently operate at a $R_{e}$ ≥ 2000 with fluid product output flows of 5 ≥ L/h [78]. In contrast, SHM and TRM microfluid chaotic advection mixing devices typically operate with an $R_{e}$ ≤ 200, with fluid product output flows ≤0.5 L/h. Although the production throughput capabilities are clearly different, both mixing processes appear to formulate quite similar mRNA LNP vaccines that can be safe and effective [48].

The significantly higher fluid flow rates and simpler design consideration for turbulent flow mixers like CIJ and MIVM mixing devices make them better suited for larger-scale clinical scale-up and manufacturing. A concise comparison of CIJ and MIVM turbulent flow mixers for mRNA LNP vaccine formulation is presented in Subraveti et al. [78]. They observed that mRNA LNP formulated with the two-jet CIJ or the four-jet MIVM configuration have very similar biophysical properties and in vitro transfection efficiencies. The four-jet MIVM turbulent flow mixing is more flexible with respect to control of the aqueous-to-ethanol flow rate ratio (FRR). This is because the flow moments of each four-jet inputs are not required to be approximately equal and balance the momentum of each fluid stream as in the case of the two-jet CIJ turbulent mixer. The MIVM mixer design allows for a lower ethanol concentration in the final post-mixture mRNA LNP solution. A lower ethanol concentration makes the ethanol removal process, e.g., dialysis or TFF, simpler compared to a two-jet CIJ turbulent mixer. However, lowering the ethanol level of the mixed fluids below 30 (*v*/*v*) % might interfere with the nanoparticle pH 7.4/ethanol fusion process that appears to be necessary to complete mRNA LNP formation [29].

Comparing strengths and weaknesses of microfluidic and turbulent flow mixing devices indicates that neither mixing process is the best choice for all mRNA LNP formulation challenges. Microfluidic mixing has a clear advantage during the lab-scale preparation of many formulation compositions for discovery screening studies. Turbulent flow mixing with CIJ or MIVN devices is better suited for the rapid, high-volume formulation needed for manufacturing-scale mRNA LNP production. The best approach would be to use microfluid mixing for lab-bench development. High-throughput (HT) multiple mRNA LNP microfluidic formulation with low sample volumes, together with sophisticated data analysis, has tremendous value for future rapid bench-scale mRNA LNP vaccine development [91]. Commercial HT low sample volume chaotic advective microfluidic technology is currently available [105,106]. Once a few lead formulation compositions are identified, scaling up to lab-scale turbulent flow mixing technology should be the priority for non-human primate pre-clinical studies. Small-scale CIJ and µMIVM mixers are commercially available for pre-clinical and early clinical scale-up [108]. Unfortunately, HT low sample volume turbulent flow mRNA LNP mixer technology for discovery research is not yet commercially available. Manufacture-scale mRNA LNP high-volume production would require the manufacture of scaled up versions of pre-clinical level CIJ or MIVM turbulent mixing devices. This was clearly performed for the manufacturing-scale production of the COVID-19 mRNA LNP vaccine Comirnaty™ [97]. The CIJ mixed mRNA LNP vaccine Comirnaty™ DLS diameter (≈100 nm) is slightly smaller than the Spikevax™ (Moderna, Cambridge, MA, USA) diameter (≈200 nm) [109,110]. Recent experience indicates that high-flow turbulent mixing is a more practical solution for clinical mRNA LNP vaccine manufacturing.

**Figure 7:**
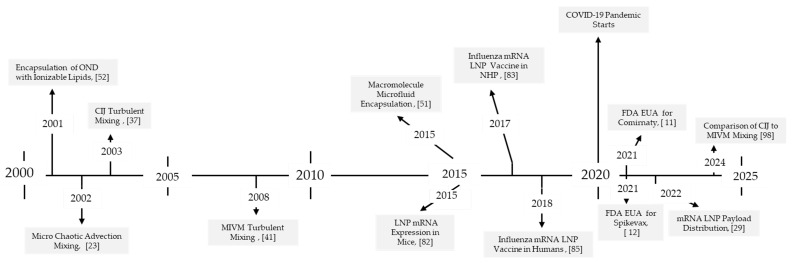
Timeline of selected significant LNP mixing-technology advances in the 21st century.

The optimal mRNA LNP vaccine delivery formulation is not yet available. The search for the optimal mixing process for each stage of mRNA vaccine formulation will continue. The timeline in Figure 7 illustrates how much has been accomplished in the last 25 years. If science can continue progress like this for the next 25 years into the 21st century, we will live in a healthier world.

## 9. Summary

The self-assembly process of the mRNA LNP vaccine is a complex process involving the chemical properties of the LNP molecules, biological processes in the target cell environment, and the solvent anti-solvent fluid mixing. The unique chemical properties of the lipids, mRNA, and buffers are probably the most crucial factors in mRNA nanoparticle self-assembly. Obviously, without the proper molecular interactions between the nanoparticle components after fluid mixing, self-assembly to a nanoparticle is not possible. This review primarily focused on the fluid-mixing principles and technologies needed for the successful mixing for the mRNA LNP vaccine formulation. Rapid and complete mixing of the lipid solvent fluid with the aqueous anti-solvent fluid phase does not necessarily ensure the self-assembly of most immunogenic mRNA LNP formulations. Cell biology and system immunology are more important to the success of a vaccine. However, rapid complete mixing by either chaotic advective microfluidics or turbulent flow will improve the reproducibility and biophysical stability properties of mRNA LNP vaccine formulations, which will enhance the possibility of clinical success.

## Figures and Tables

**Table 1:** Summary of individual nanoparticle types after micromixing (pH 4.0) and dialysis buffer exchange (pH 7.4) was quantified by fluorescent CICS. From Li et al., “Payload distribution and capacity of mRNA lipid nanoparticles” [29].

Process Condition	Fluorescently Identified Particle	Population by Number	mRNA Copy per Particle	Average mRNA EE %	Average DLS Diameter, nm	Average ζ-Potential, mV
Initial LNP, post-T-mixing 3:1, aqueous/ethanol (*v*/*v*) mixed solution, pH 4.0	Lipophilic mRNA complex (LNC)	34 ± 8%	3.4 ± 0.4	N/A	106 ± 13	+45 ± 1
	Non-lipophilic mRNA complex (NLNC)	25 ± 4%	1.3 ± 0.2			
	mRNA lipid nanoparticle (LNP)	0%	N/A			
	Empty particles, i.e., no mRNA (ENP)	41 ± 10%	0			
Final-process LNP, post-dialysis aqueous pH 7.4 buffer	Lipophilic mRNA complex (LNC)	0	N/A	94.2 ± 0.4%	120 ± 6	−6.3 ± 1.3
	Non-lipophilic mRNA complex (NLNC)	0	N/A			
	mRNA lipid nanoparticle (LNP)	23 ± 8%	2.8 ± 0.4			
	Empty particles, i.e., no mRNA (ENP)	77 ± 8%	N/A			

**Table 2:** Comparison of LNP formulation properties prepared by different mixing processes. From Subraveti et al., “Synthesizing Lipid Nanoparticles by Turbulent Flow in Confined Impinging Jet Mixers” [78]. * Encapsulation efficiency.

Mixing Method	DLS Diameter, nm	ζ-Potential, mV	mRNA EE % *	HeLa Cell In Vitro Transfection, RLU/1000 Cells	HeLa Cell Viability
Manual pipette	≈140	≈0.5	>95%	≈15	≈100%
Two-jet CIJ mixer	≈90	≈2.0	>95%	≈100	≈100%
Four-jet MIVM	≈90	≈1.0	>95%	≈100	≈100%

**Table 3:** Pharmaceutical-oriented experiment information about buffers and LNP compositions used in selected nanoparticle-formulation studies.

Reference	Mixing Technology	Lipids (Total mg/mL)	Lipid Solvent	RNA Type (mg/mL)	mRNA or si RNA Buffer	Dialysis Buffer	Diameter, nm	LNP EE%	Comments
2013, Maier, M.A., et al. [68]	TJM	L13:DSP:chol:PEG-DMG 55:10:32:2 mole ratio	Ethanol	Si RNA 1 mg/mL	10 mM Citrate pH 4.0	PBS pH 7.5	≈60	>90%	Demonstrate in vitro and in vivo potency
2015, Leung, A.K., et al. [51]	SHM	DLIN-KC2-DMA:DSPC:Chol:PEG-lipid 40:11:47:1 mol ratio (xxx)	Ethanol	1.7 kbp siRNA (yyy)	25 mM Na Acetate pH 4.0	50 mM MES/50 mM Na Citrate pH 6.7 -> PBS pH 7.4	≈50	90%	Evaluated many mixing conditions using Cryo-TM
2017, Richner, J. M., et al. [85]	SHM	ionizable lipid:DSPC:cholesterol:PEG-lipid 50:10:38:2 mole ratio	Ethanol	preM-E Zika mRNA	50 mM citrate buffer pH 4.0	PBS pH 7.5	80–100	>90%	Induced protective antibodies in mice
2017, Liang, F., et al. [84]	SHM	ionizable lipid:DSPC:cholesterol:PEG-lipid:GLA 50:10:38:2:0.2 mole ratio	Ethanol	HA influenza mRNA	50 mM citrate buffer pH 4.0	PBS pH 7.4	80–100	>95%	NHP Exaluation of mRNA vaccine
2017, Bahl, K., et al. [86]	SHM	ionizable lipid:DSPC: cholesterol: PEG-lipid 50:10:38.5:1.5 mole ratio	Ethanol	HA influenza mRNA	50 mM citrate buffer pH 4.0	PBS pH 7.4	80–100	>90%	Clinical Exaluation of mRNA vaccine
2018, Pardi, N., et al. [83]	SHM	ionizable cationic lipid/ phosphatidylcholine/cholesterol/PEG-lipid 50:10:38.5:1.5 mole ratio	Ethanol	HA influenza mRNA	50 mM citrate buffer pH 4.0	PBS pH 7.4	80	>90%	Induces HA influenza anti-stalk antibodies in mice
2020, Ripoll, M., et al. [30]	TRM	MC3:DOPC: cholesterol:PEG-lipid 50:10:38.5:1.5 mole ratio	Ethanol	pDNA	50 mM citrate pH 4.0	PBS pH 7.4	100	≈ 80	Evaluated many mixing conditions using Cryo-TM
2924, Subraveti, S. N., et al. [78]	CJI	MC3:DSPC: cholesterol:DMG-PEG2000 50:10:38.5:1.5 mole ratio	Ethanol	Yeast mRNA	20 mM Acetate pH 4	10 mM Hepes pH 7.5	≈90	>95%	Input jet 1 -> lipids Input jet 2 -> RNA
2924, Subraveti, S. N., et al. [78]	MIVM	MC3:DSPC: cholesterol:DMG-PEG2000 50:10:38.5:1.5 mole ratio	Ethanol	Yeast mRNA	20 mM Acetate pH 4	10 mM Hepes pH 7.5	≈90	>95%	Input jets 1 & 3 -> lipids Input jets 2 & 4 -> RNA

**Table 4:** (a) Information about microfluidic mixers suitable for mRNA LNP formulation development currently commercially available (b) Information about turbulent mixers suitable for mRNA LNP formulation development currently commercially available.

(a)			
Product Trade Name	General Comments	Vendor	Website
NanoAssemblr™ Spark™	Small-scale rapid formulation screening 25 to 250 µL batch volume	Cytiva	https://www.cytivalifesciences.com/en/us/shop/lipid-nanoparticle-instruments-and-reagents/nanoparticle-formulation-systems?sort=NameAsc&chunk=1 (accessed on 13 July 2025)
NanoAssemblr™ Ignite™	Pre-clinical formulation screening, easy to use TRM, 5 to 60 mL batch		
NanoAssemblr™ Blaze™	Large-scale for process scale-up, includes TFF 0.2 to 10 L batch		
NanoAssemblr™ GMP System	GMP system for clinical supplies 1 to 50 L batch size		
NanoAssemblr™ commercial formulation system	GMP system for large-scale commercial manufacturing		
Tamara	Easy to use, reusable microchips 0.2 to 30 mL batch size	Inside Therapeutics	https://insidetx.com/product/tamara/ (accessed on 13 July 2025)
Lipid nanoparticle synthesis pack	Pressure controlled SHM, easy to use 0.5 mL to 5 L batch size	Elve Flow	https://www.elveflow.com/microfluidics-application-packs/lipid-nanoparticle-synthesis/ (accessed on 13 July 2025)
NanoGenerator™ Flex-S	Small-scale discovery screening, multi-sample 1 to 4, 0.1 to 0.5 mL per sample	PreciGenome LLC	https://www.precigenome.com/ (accessed on 13 July 2025)
NanoGenerator™ Flex-S Plus	Early discovery, fully automated HT, multi-sample 1 to 96, 0.1 to 0. mL per sample		
NanoGenerator™ Flex-M	Pre-clinical formulation, in-line ethanol dilution 1 to 12 mL batch		
NanoGenerator™ MAX +	cGMP system for clinical manufacturing, product throughput > 10 L/h.		
Sunscreen	Discovery, microfluid chip options, automated HT, 1 to 96 samples, 0.2 to 2.0 mL per sample	Unchained Labs	https://www.unchainedlabs.com/lipid-nanoparticles/ (accessed on 13 July 2025)
Sunshine	Pre-clinical, microfluid chip options, automated 1 to 10 samples, continuous flow up to 30 mL/min		
Sunbather	GMP Clinical ready, microfluid chip options, continuous flow up to 1.8 L/h.		
**(b)**			
**Product Trade Name**	**General Comments**	**Vendor**	**Website**
DIANT^®^ LARU Discovery	Discovery-scale continuous turbulent jet mixing 2 mL minimum output volume	Diant Pharma Inc.	https://diantpharma.com/ (accessed on 13 July 2025)
DIANT^®^ LARU—Benchtop	Pilot-scale continuous turbulent jet mixing with TFF and PAT max output 0.4 L/min		
DIANT^®^ LiFT—HT	Commercial-scale GMP continuous turbulent jet mixing with TFF and PAT max output 20 L/min		
Nova™ Benchtop	Discovery-scale CIJ mixer system in-line dilution TFR 0.1 to 100 mL/min	Helix Biotech Inc.	https://www.helixbiotech.com/ (accessed on 13 July 2025)
Platform for Intracelluar Delivery of DNA & RNA	Discovery-scale turbulent mixing technology for intracellular RNA and DNA delivery	Optimeos Life Sciences Inc.	https://optimeos.com/ (accessed on 13 July 2025)
CIJ & MIVM Mixers Design by Dr. Prud’homme’s Princeton Lab	Manufactures CIJ and MIVM turbulent mixer units for lab-scale formulation development	Holland Applied Technologies	https://hollandapt.com/products/fittings-components/cij-mivm-mixers/ (accessed on 13 July 2025)

## Abbreviations

The following abbreviations are used in this manuscript:

BM	Microfluidic Baffles Mixer Design
CICS	Cylindrical illumination confocal spectroscopy
CIJ	Confined Impinging Jet Turbulent Flow Mixer
CFD	Computational fluid dynamics
DMP	Dimethyloxypropane
FNC	Flash nanocomplexation (of nanoparticles)
FNP	Flash nanoprecipitation (of nanoparticles)
FRR	Flow rate ratio
HFFM	Hydrodynamic Flow-Focusing Mixer Design
LNP	Lipid nanoparticle
MIVM	Multi-inlet vortex turbulent flow mixer
SHM	Staggered Herringbone Mixer Design
TFR	Total flow rate
TJM	T-junction mixer design
TRM	Toroidal ring mixer design
